# Implantation of a Cardiac resynchronization therapy system in a patient with an unroofed coronary sinus

**DOI:** 10.1002/joa3.12401

**Published:** 2020-07-20

**Authors:** Violeta Groudeva, Svetoslav Iovev

**Affiliations:** ^1^ Department of Diagnostic Imaging Saint Ekaterina” University Multiprofile Hospital for Active Treatment Medical University Sofia Sofia Bulgaria

**Keywords:** computed tomography, congenital heart disease, coronary sinus atresia, unroofed coronary sinus

## Abstract

The unroofed coronary sinus (URCS) is a spectrum of cardiac anomalies in which part or all of the common wall between the coronary sinus and the left atrium is absent. Rarely, it is associated with coronary sinus atresia. The diagnosis of this lesion is important for the prognosis of the patient, especially in cases when cardiac interventions such as CRT implantation needs to be performed. It is found incidentally because of nonspecific clinical features. We report a case of a complete URCS and CS atresia during a computed tomographic investigation performed following prior impossibility of LV lead to be implanted.

## Graphical abstract

This case report proves the necessity of cardiac imaging examination prior cardiovascular procedure such as CRT implantation.

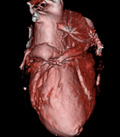

## 1 INTRODUCTION

Unroofed coronary sinus is a rare congenital cardiac anomaly in which there is partial (either focal or fenestrated) or complete absence of the roof of the coronary sinus, which results in a communication between the coronary sinus and the left atrium. Unroofed coronary sinus is the rarest type of atrial septal defect. It is often associated with persistent left superior vena cava and other forms of complex congenital heart disease, usually heterotaxia syndromes.^1^

Although unroofed CS and CS atresia often remain silent due to nonspecific clinical features, the diagnosis is difficult but important as it can cause impediments during intravenous interventions such as CRT implantation, as it happened in our case.

Cardiac resynchronization therapy is an adjunct current therapy in patients with systolic heart failure and prolonged interventricular conduction^2^ aiming to restore the normal synchronization between the two ventricles. This can be achieved by implantation of a pacing lead in a CS branch draining the free lateral wall of the LV. Unfavorable anatomy impairs a successful procedure.

## 2 CASE REPORT

**FIGURE 1:**
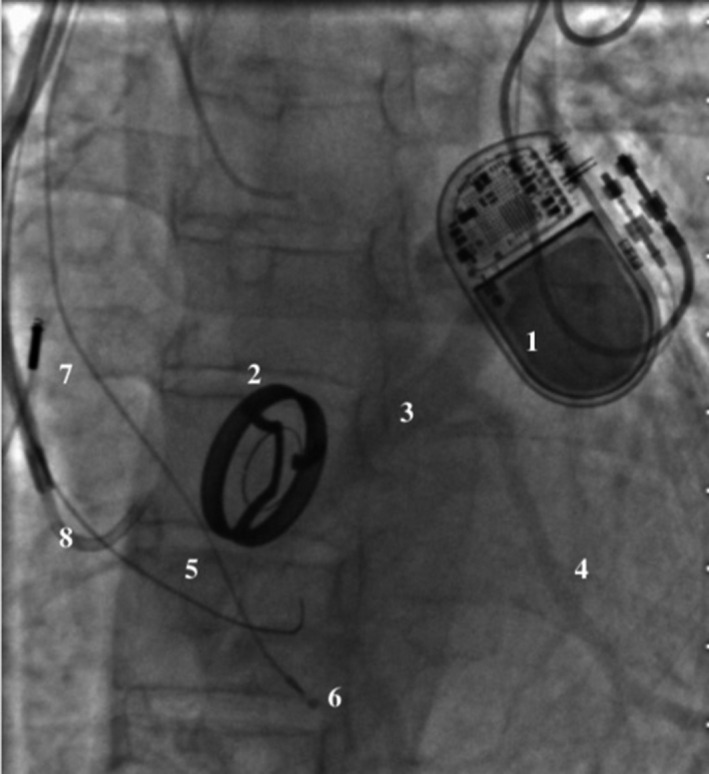
Retrograde angiography of the coronary sinus. 1. Two‐cavity pacemaker—DDD R. 2. Mitral valve. 3. Great cardiac vein. 4. Posterolateral branch of the coronary sinus. 5. Ostium of the coronary sinus. 6. Unipolar lead in the right ventricle. 7. Bipolar lead in the right atrium.. 8. Introducer for coronary sinus intubation

**FIGURE 2:**
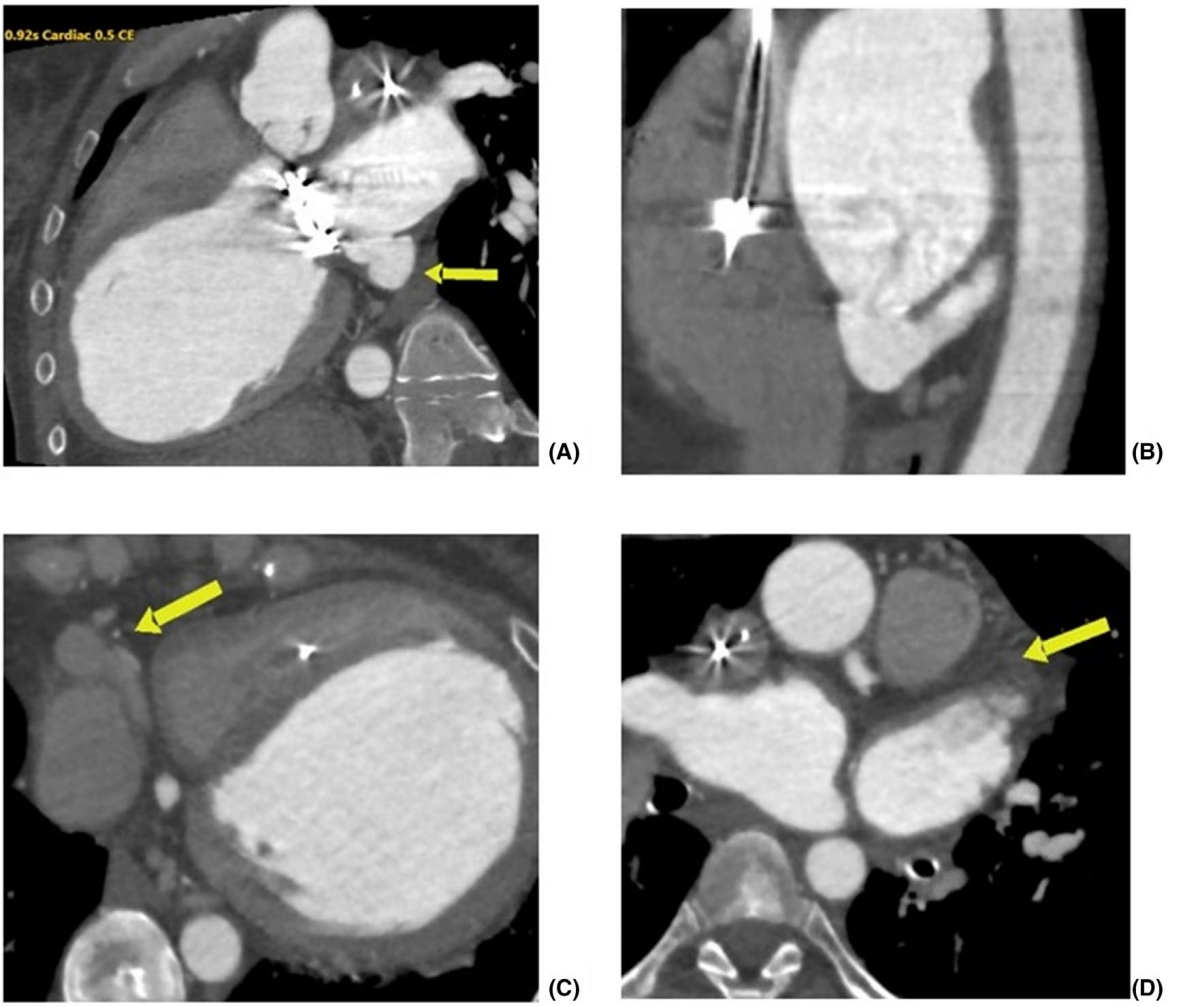
Computed tomography angiography (CTA), oblique multiplanar reconstructions. (A) Unroofed coronary sinus (yellow arrow), wide communication with left atrium. (B) Unroofed coronary sinus with abrupt discontinuation. No connection with right atrium is visible. (C) Enlarged cardiac veins draining into the right atrium (yellow arrow). (d) axial plane, no persistent left superior vena cava is present (yellow arrow)

We present a 37‐year‐old Caucasian female referred to our hospital for cardiac resynchronization therapy due to left ventricle dysfunction. The patient had a history of congenital atrioventricular septal defect which was repaired surgically at the age of 2. Limited medical records had been available. At the age of 8, the patient underwent mitral valve repair. Postoperative complete AV block was present which required implantation of a pacemaker. The patient came to the clinic with complains of exertional dyspnea at minimal physical activities. Holter ECG proved short episode of ventricle tachycardia. Concomitant arterial hypertension with poor control was also present. Transthoracic echocardiography revealed dilated globular left ventricle, with diffusely decreased ejection fraction/ EF 34%/ and septal dyskinesia. End diastolic volume was of 195 mL, and end systolic volume of 125 mL. Peak diastolic gradient of the mechanic mitral valve prosthesis was 11 mm/Hg. Aortic valve was with normal function. Normal right heart chambers with indirect systolic right ventricle pressure 30 mm/Hg. The patient was referred for cardiac resynchronization therapy. During the procedure, the coronary sinus was not possible to be cannulated and only right ventricular defibrillation lead was implanted (Figure 1). Therefore, the patient was referred for computer tomography. Gated cardiac CT was performed with Aquillion One, Toshiba machine, following a standard protocol. (Figure 2) The CT showed normal anatomy of coronary arteries. Coronary sinus was visualized in the posterior atrioventricular groove measuring 12 mm. Toward the right atrium the sinus was with saccular dilatation and then stopped abruptly without connection with the right atrium. Along the upper border of the terminal part of the sinus, a defect measuring 15 mm was noted and a communication with the lying cranially left atrium. A small vessel measuring 1.5 mm in diameter connected the coronary sinus with the right atrium was detected. Dilated anterior cardiac veins drained into the anterior wall of the right atrium. Also, numerous tortuous small venous vessels were seen in anterior wall of the heart draining into the right atrium and ventricle. No persistent superior vena cava was presented.

**FIGURE 3:**
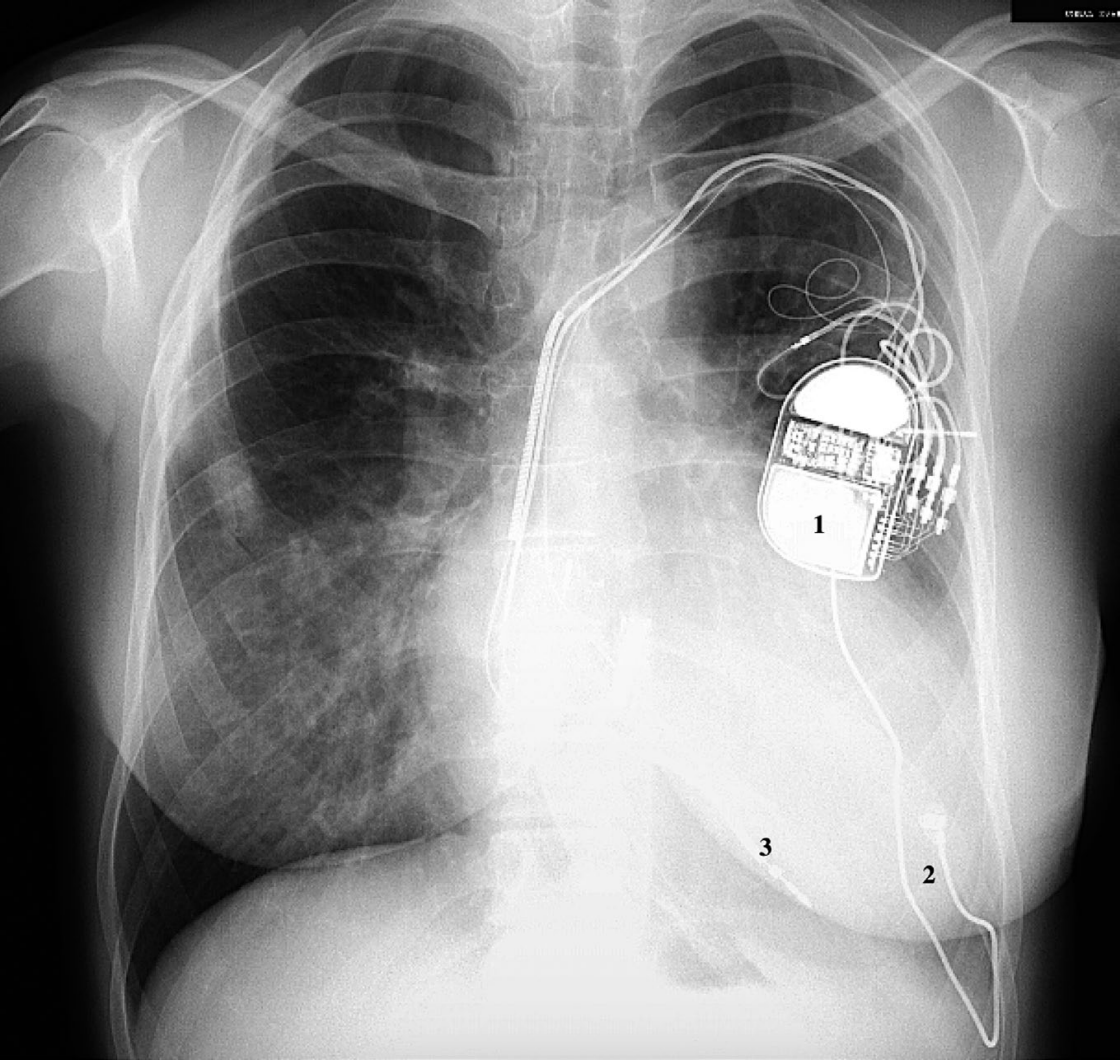
Chest X‐ray after surgical implantation of epicardial lead. 1. CRT‐D Device. 2. Left ventricular (LV)—epicardial lead. 3. Right ventricular (RV)—defibrillation lead

Due to lack of accessibility to cannulate the coronary sinus and the entire cardiac venous system, the patient was referred for surgical lead implantation (Figure 3).

## 3 DISCUSSION

Unroofed coronary sinus occurs when a communication between the coronary sinus and left atrium exists, which results in aberrant drainage of the cardiac veins into the left atrium.

The morphological types have been classified into four groups: type I—completely unroofed with PLSVC; type II—completely unroofed without PLSVC; type III—partially unroofed mid portion; and finally type IV—partially unroofed terminal portion as illustrated in the present case.^3^ Also, in our case, the unroofed CS is accompanied by coronary sinus ostial atresia.

The diagnosis of coronary sinus abnormalities is not straightforward. The coronary sinus dilatation can be easily visualized by transthoracic and trans‐esophageal echocardiography. However, detection of defect between coronary sinus and left atrium by echocardiography can be quite challenging due to limited sonographic window. In our case, transthoracic echocardiography did not depict the coronary sinus defect. Moreover, the patient underwent previous cardiac surgeries that did not document coronary sinus atresia, neither unroofing of the coronary sinus. Omission of coronary atresia even after cardiac surgery has been previously reported in literature.

The clinical presentation depends on the size of the communication between coronary sinus and left atrium and the degree of left to right and right to left shunt. Often, they remain silent as in our case. When it is symptomatic, the symptoms may range from mild, nonspecific complaints to severe dyspnea with symptoms of right‐sided heart failure from chronic right ventricular volume overload. In case of right to left shunt, a potential complication of brain abscess and emboli exists.

Most cases of CS atresia are associated with an alternative exit for coronary venous blood return, such as a PLSVC, large Thebesian vein, or CS canal defect.^4^ In our case, there are collateral small venous pathways. However, no persistent left superior vein was present which can be used as an alternative path for LV lead implantation. In such cases, an optional treatment is surgical epimyocardial lead implantation.

## 4 CONCLUSION

A thorough knowledge of the cardiac venous anatomy prior to the procedure will facilitate the intervention process and increase the successful outcome.

Cardiac CT with its excellent spatial resolution allows for the accurate morphological evaluation of the structures of the heart.

## CONFLICT OF INTEREST

The authors declare no conflict of interest.
